# Topical Fluoride Applications Related Posts Analysis on Twitter Using Natural Language Processing

**DOI:** 10.3290/j.ohpd.b2048359

**Published:** 2021-09-22

**Authors:** Basak Kiziltan Eliacik

**Affiliations:** a Assistant Professor, Department of Pediatric Dentistry, Hamidiye Faculty of Dental Medicine, University of Health Sciences, Istanbul, Turkey. Study conception and design; acquisition, analysis and interpretation of data; drafting of the manuscript.

**Keywords:** dental caries, topical fluoride, public health, social media, Twitter, natural language processing technique

## Abstract

**Purpose:**

Social media is today a comprehensive source of data that can serve as a guide to professionals in issues related to public health. The purpose of this paper is to investigate the content of topical fluoride-related Twitter posts made over a 3-year period in order to improve our understanding of Twitter users’ perceptions and treatment experiences.

**Materials and Methods:**

A continuous cross-sectional sample of Tweets on the subject of ‘approaches to the topical fluoride treatment of tooth decay’ was collected from the Twitter social networking platform between 1 January 2017 and 1 January 2020 using a software application developed for this research that makes use of the Twitter advanced search API. The words and phrases used for the identification of related Tweets were determined through a screening of the topical fluoride keywords of previous studies, and a search was conducted in the English language. To better arrange the collected Tweets and to make the data more meaningful, firstly one of the natural language process techniques – Tokenization – was applied, after which the Tweets were converted into a set of meaningful words and regular expressions. The Tweets were then compared with each other, word-by-word, with the help of a word-based Levenshtein distance algorithm, after which two experts in the computational social science domain labelled each Tweet.

**Results:**

A total of 132,358 Tweeter posts referencing topical fluoride applications were collected, of which 110,847 were eliminated through the use of a word-based Levenshtein distance algorithm, and the remaining corpus of 21,511 posts was analysed and evaluated for specific content. Within the garnered data, 48.5% (n = 10,428) of the Tweeter posts concerned topical fluoride treatments, and 7% (n = 1,507) reported experiences with topical fluoride treatment. Negative Tweeter posts about topical fluoride treatment (5,679, 26.4%) vastly outnumbered those that were positive (3,897, 18.1%).

**Conclusion:**

The current study achieved its main objectives of analysing topical fluoride application-related posts made on social media. From the garnered Twitter data, it can be understood that Twitter users regularly share their concerns and negative sentiments about the side effects of topical fluoride treatments on the platform. Future explorations of social media may aid public health and dental professionals in the development of strategies to educate the public and to raise awareness of the importance of topical fluoride applications.

Tooth decay is recognised as one of the leading public health problems in every country, where oral hygiene control, fluoride and fissure sealant protocols are used to reduce instances of caries.^6, 16^ In socially disadvantaged populations, severe early childhood caries (ECC) are a major oral health problem, the prevalence of which varies worldwide.^23^ Even though most children are able to undergo standard dental treatment in conventional settings, general anaesthesia (GA) may be needed for the more uncooperative and emotional children in early childhood. An increase has been noted in the use of GA for paediatric dental treatment due to the benefits it offers to patient comfort and safety during medical procedures, although it comes with a statistically significant risk of morbidity and occasionally mortality, while also increasing healthcare costs.^8^ As an alternative, topical fluoride treatment, which is known to inhibit the formation of caries by increasing remineralisation and preventing enamel demineralisation, is a simple and cheap approach that is thought to reduce significant incidences of severe ECC, and consequently, the rate of dental treatments under GA.^21^

Although the efficacy of fluoride in the prevention of caries has been proven in literature, there is a lack of consensus on the use of topical fluoride in public discussions.^1, 14, 19^ Recent studies have shown that 13–22% of parents refuse fluoride treatments for their children during preventive dental visits, and even more parents believe there to be unresolved concerns related to topical fluoride applications.^7, 15, 22, 27^ Fluoride refusal is a growing phenomenon that can often be attributed to parental decisions based on concerns about the necessity, safety or consequences of fluoride applications.^6^ Studies to date assessing the public opinion of fluoride treatments has been limited to questionnaire studies.^4–6, 12, 15, 22^ In a survey study conducted by Hendaus et al investigating parental preferences as regards to fluoride polish, it was found that over 90% of the participants were aware that oral and dental health affects the whole body, and that nearly 70% of parents were unaware of the existence of fluoride varnish. The study also found that 40% of the participants had concerns about the treatment, as there was a risk the children could swallow the polish.^15^ Similar to this study, Carpiano et al carried out a pre-tested survey of parents who declined topical fluoride treatment for their children,^4^ and found that 51.5% had refused fluoride treatment due to concerns about side effects and safety. Although 16.9% of the study group stated that they had obtained information about fluoride from the Internet, their reasons for refusal remained unclear. Before we can work on changing such negative attitudes toward fluoride in parents, it is first necessary to understand the behavioural, cultural and social origins of fluoride refusal behaviours.

While public trends on a given subject have historically been determined through surveys, in the present day, such trends can be better understood from social media platforms, given the intensive use of social media as a communication tool. The social media platform Twitter has 320 million active monthly users, being a platform from which people are able to instantly express their thoughts and feelings. The ability to share one’s thoughts and feelings as a Tweet on a public platform has turned Twitter into a unique real-time source of information for researchers.^25^

To the best of our knowledge, this current study is the first attempted content analysis of Twitter in relation to topical fluoride treatments. The purpose of this paper is to investigate the content of topical fluoride-related Twitter posts made over a 3-year period in order to improve our understanding of Twitter users’ perceptions and treatment experiences.

## Materials and Methods

The present study analyses the content of Tweets about topical fluoride treatments, making use of a software program that uses a word-based Levenshtein distance algorithm to improve our understanding of the attitudes, thoughts and treatment experiences of Twitter users. A continuous cross-sectional sample of Tweets was collected using a software application developed specifically for the research (by A.B.E) that makes use of the Twitter advanced search API. Tweets made between 1 January 2017 and 1 January 2020 mentioning approaches to the topical fluoride treatment of tooth decay were searched from the Twitter social networking platform using words and phrases garnered from the keywords of topical fluoride studies. The determined words were: fluoride, tooth decay, child tooth, pedodontics, paediatric dentistry, fluoride varnish, fluoride polish, teeth fluoride, tooth fluoride, toothpaste fluoride, teeth varnish, tooth varnish, topical fluoride, pineal calcification and caries risk. Included in the analysis were any Tweets containing at least one of the keywords. The searches were made in the English language. All of the Tweets obtained during the search were saved in separate files for each quarter (3 months).

The collected Tweets were unsuitable for immediate analysis, as the study was focused on people’s personal thoughts about topical fluoride. Consequently, all non-human Tweets, such as advertisements and software bot re-Tweets, were removed from the sample.

To tidy up the collected Tweets and to make the data more meaningful, firstly, a natural language processing technique, Tokenization, was applied, after which the Tweets were converted into a set of meaningful words and regular expressions. All Tweets were then compared word-by-word with the help of a word-based Levenshtein distance algorithm. Re-Tweets are treated the same as original Tweets, and advertisements’ contents are similar to each other. The data mining cleaning phase led to the automatic exclusion of re-Tweets by software bots and advertisements with similar content. All Tweets in this study were original posts voicing concern, experience, negative and positive thoughts.

In the next stage, two experts (ABE and TY) in computational social science aided in the labelling of the Twitter data, analysing 500 randomly selected Tweets and assigning them to four different categories. If the experts agreed on which category the Tweet belonged to, the Tweet was included in the comparison sets. This labelling approach led to the creation of a Tweet data set containing Tweets about professional and self-applied topical fluoride treatments, and Tweet entries voicing concerns (category A, n = 215), Tweet entries voicing experiences (category B, n = 43), Tweet entries making positive thoughts (category C, n = 92) category C, and Tweet entries making negative thoughts (category D, n = 150). The inter-rater reliability of the collected data was evaluated through the calculation of the percentage agreement and the kappa for each subcategory.

Next, the data were compared with the remaining components of the data sets to identify the categories of the other Tweets using a word-based Levenshtein distance algorithm. The syntactic similarity of each Tweet with the Tweets in the four categories was calculated, and the Tweet was subsequently added to the category with the most syntactic similarity. Thus, four different categories were formed.

We then used TwiRole to identify the gender of the Twitter users.^18^ TwiRole is a hybrid model for user classification on Twitter of men, women and brands. The TwiRole model is implemented using Python, and its implementation is downloadable from the GitHub repository (https://github.com/liuqingli/TwiRole)

The statistical analyses were carried out using IBM SPSS Statistics for Windows (Version 25.0. Armonk, NY: IBM Corp.). Chi-square tests were applied to identify any statistically significant differences in the distribution of Tweets between genders, and a run test was used to analyse the changes in number of Tweets in each category over the 3-year study period. Between-group differences were considered to be statistically significant at p <0.05.

## Results

**Fig 1:**
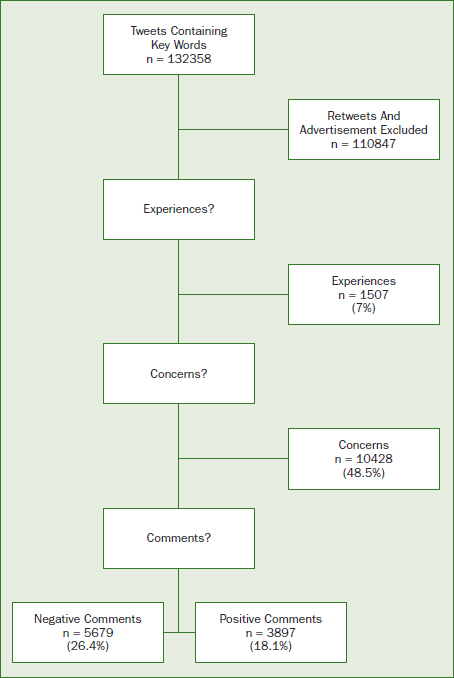
Flowchart of the classification sequence for the qualitative analysis.

**Fig 2:**
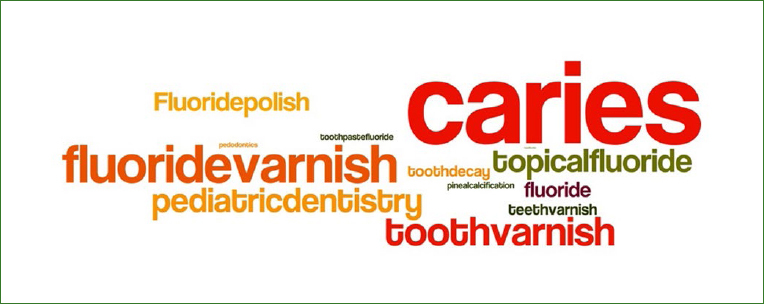
Word cloud created from Tweet content for the 21,511 selected Tweets. The larger the font, the greater the frequency of the word appearing in Tweets related to topical fluoride.

Of the 132,358 Tweets collected, 110,847 were eliminated after the application of a word-based Levenshtein distance algorithm, and 21,511 Tweeter posts from 21,511 unique user were included in the study. An average of 1,792 Tweets were collected each quarter containing the searched keywords (standard deviation = 409.23, max = 2,462, min = 1,300, median = 1,735.5). The most frequently used topical fluoride hashtags were: #caries (8,627 Tweets), #fluoridevarnish (4,823 Tweets), #toothvarnish (3,441 Tweets), #topicalfluoride (1,258 Tweets) and #pediatricdentistry (1,021 Tweets) (see Figs 1 and 2).

When the Tweets were examined for syntactic similarity, it was found that 48.5% (n = 10,428) posted concerns about topical fluoride treatment and 7% (n = 1,507) highlighted problems experienced during and after topical fluoride administration. The frequency of positive to negative posts related to topical fluoride prophylactic treatment was 18.1% (n = 3,897) and 26.4% (n = 5,679), respectively. Following are some examples for randomly selected 500 posts used to labelling and analysing of the Twitter data (Table 1):

**Table 1:** Examples of concern, experience, negative and positive posts on Twitter

Tweet category	N (% of Tweets)	Illustrative examples^1^	Per cent agreement	Kappa value
Posts voicing concern about topical fluoride treatments	10,478 (48.5%)	If fluoride decreases children’s IQ, are harder teeth worth the risk?	0.92	0.93
Experiences of topical fluoride treatment	1,507 (7%)	If I ranked the taste of dental Fluoride, it would rank below the fifth minute of Double Bubble gum but above V8 Juice, approximately	0.94	0.96
Positive posts on topical fluoride treatment	3,897 (18.1%)	Fluoride-based toothpaste and mouthwashes are scientifically proven to reduce the risk of cavities by 20–30%	0.84	0.92
Negative posts on topical fluoride treatment	5,679 (26.4%)	The authors conclude that available evidence suggests that fluoride has the potential to cause major adverse human health problems, while providing only a modest level of prevention against dental caries (cavities)	0.87	0.91

^1^ While all Tweets included in the analysis were posted publicly, to protect the privacy of those who posted the Tweets, we have paraphrased the wording of the Tweets to ensure anonymity without changing the meaning.

Concerns – P23: ‘If fluoride decreases children’s IQ, are harder teeth worth the risk?’

Negative – P121: ‘Cancer caused only 4% of deaths to Americans in 1900. This was before vaccines, GM food, artificial sweeteners & dyes, preservatives, rBGH, fluoride, & trans fats. Now ~40% will develop cancer & 20% will die. 400% increase in cancer deaths from 1900. END the #FDA & #CDC’.

Positive – P47: ‘Done fluoride varnish for Emily. I was so happy when the doctor praised her teeth’.

**Fig 3:**
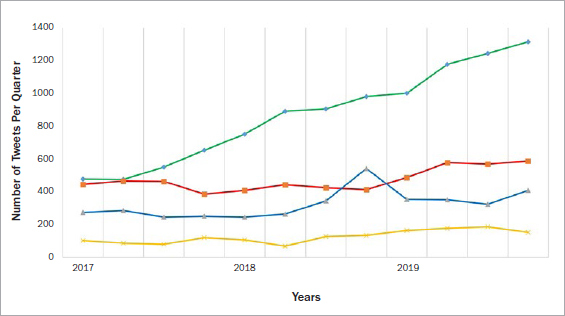
Changes in the number of Tweets in each category per quarter (3-month period). Green line: Posts about concern; red line: negative posts; blue line: positive posts; yellow line: posts about experience.

The number of Tweets in the concern category was 478 in the first quarter of 2017, and 1,315 Tweets in the last quarter of 2019. During the study period, negative Tweets saw only a limited increase in number, despite the statistically significant increase in the number of concern Tweets over the past three years (see Fig 3). Using TwiRole, the gender of Twitter users could be identified in 87% (18,715/ 21,511) of cases, revealing 75% of the Tweets to have been made by females and 12% by males. The remaining 13% of Tweets were from accounts in which the gender was not determined. While Tweets reporting concern (n = 6,987, 67%) or experiences (874, 58%) were mostly shared by females, the gender difference was statistically significant only in the former (p <0.01).

## Discussion

A total of 21,511 Twitter posts that were analysed provided some insights about the perceptions of Twitter users and their experiences over a 3-year period. An average of over 1,700 fluoride-related Tweets were collected each quarter, and it was found that the majority of shared Tweets voiced concerns about topical fluoride, with a statistically significant difference identified between genders in terms of the number of Tweets voicing concern.

Previous survey studies represented a valuable source of information that can be used to evaluate the public’s perception of the topical fluoride treatments. Most of these studies concluded that parental knowledge and attitudes toward topical fluoride polish had a negative effect on their children’s dental health.^4–6, 15^ In contrast to the results of these studies, Ferraioli et al reported that although 24% of the respondents in their study were concerned about the side effects of fluoride, 96% took a positive view of fluoride treatment. Furthermore, 84% of the respondents in the study believed oral and dental health to be closely related to general health.^12^ Although the results of the present study were partially consistent with those of earlier survey studies on this subject, there were some differences in the results. In the present study, the percentage of those concerned about topical fluoride treatment was found to be 48.5% – similar to the findings reported by Hendaus et al and Carpiano et al – but when negative posts were taken into account, it was observed that 74.9% of Twitter users in the study group were cautious about fluoride treatments.

Earlier studies have reported that those with concerns about fluoride applications, and who thus refuse such treatments, are mostly the parents of children with autism spectrum disorder, with rejection rates being higher among parents under the age of 35 years and university graduates. In addition to the results of the above studies, it was noted in the present study that Tweets voicing concerns and experiences with topical fluoride treatments were mostly shared by women, while there was no difference in gender among those voicing positive or negative opinions. As a result, this study shows that if concerns about topical fluoride are to be eliminated, the greatest benefit would be gained from informing the female relatives of children about the preventive and real side effects of this treatment.

Although these papers did not clearly define the reasons for rejection, the results of previous epidemiological studies were given. Accordingly, the main concern about topical fluoride applications was that increased fluoride intake can lead to IQ deficiency in children, especially in the early development period, due to its neurotoxic effects, along with other systemic childhood conditions.^26^ This idea has been supported by social media groups and some academic publications in literature.^11, 13, 17, 20, 28^ In the present study, when examining Tweets with posts with negative thoughts and concerns, it was observed that the most frequently discussed issue was the negative effects of fluoride on IQ.

Chi et al made a retrospective examination of those who rejected fluoride applications when recommended during an oral examination, establishing a rate of 4.9% in their review of clinical files, while in the second part of the study, the authors administered a questionnaire to determine rejection rates, and reported a rate of 14.6%. In the present study, the rate of Tweets expressing concerns and negative thoughts was 74.9%.

When the results of the above studies are considered, it can be understood that people may be reluctant to give their true opinions in a one-to-one application of a questionnaire, that individuals who reject fluoride are considered hesitant, but that not all hesitant individuals reject fluoride treatments. There is a risk that those who are on the fence will eventually join the group of fluoride refusers, and it can be concluded that social media, where people are able to express their opinions freely, is better able to reveal the difference between the two. Disinformation on the Internet may be an important factor in the increase in parental refusal rates for this treatment.

Although the information obtained on a given subject through survey studies is considered to be of higher quality, as well as more relevant and more precise, all such studies have their limitations. In these types of studies, however, the researcher can gain deeper insight from specific answers by treating the questionnaire like a meaningful discussion and deducing the validity of each response. In addition to their inherent disadvantages, survey studies are conducted within a limited timeframe, while nowadays, the usefulness of social media in medical studies and as a social platform is emerging as a new area of study. Social media offers the advantages of allowing information about the research question to be obtained from the general public and permits researches to be carried out retrospectively. The present study can be considered unique in its discussion of the change in public perception of fluoride practices over time, which is absent from other studies carried out to date. In this regard, a marked increase was noted in the distribution of shared Tweets containing negative thoughts and concerns about fluoride applications over a three-year period, from 2017 to 2020. With the rapid increase in social media use, the growing phenomenon of fluoride avoidance has become a public health problem. Unconfirmed assumptions about topical fluoride applications on the Internet trigger negative thoughts about such treatment approaches in the minds of parents. Conversely, while fewer positive Tweets were posted prior to the last quarter of 2018, a statistically significant upward surge was noted in positive Tweets that reached its highest point in this quarter, coinciding with the “I love fluoride” campaign of ADA.^2^ This increase had been started by the middle of 2018 and remarkably stable for more than 3 months, suggesting policies and campaigns that support fluoride applications implemented by some organisations and governments might play an important role in the increase of posts with positive thoughts.^3, 9, 10, 24^

Although we accessed a sizeable quantity of Twitter conversations, our findings cannot be generalised to all social media users. The known limitations associated with the use of social media for research also apply to the present study, including the sporadic nature of shares made on social media, the potential for misinterpretation of the message content, and the brief nature of the messages. Further studies should be carried out on social media users that may result in a higher number of respondents, and thereby the possibility of an even more detailed analysis based on a further sub-categorisation of the respondents. In future studies, collecting data from a longer period and using more keywords may provide more detailed results, while expanding the study to other social media platforms and comparing and/or combining the results with those garnered from more traditional approaches may increase the reliability of the findings.

## Conclusion

This is the first study to analyse topical fluoride treatments related posts made on Twitter using natural language processing. Our findings suggest that Twitter users use social media sites such as Twitter to convey mostly concern and negative feelings about fluoride. As seen in this study, to counterbalance these negative thoughts with more positive thoughts, there is a need for public health researchers to develop fluoride refusal screening tools and evidence-based strategies aimed at educating the public on the importance of topical fluoride applications.
